# The Structure, Assembly Processes of Microbial Communities and Their Effects on the Quality of Goat MEAT During Chilled Storage (4 °C)

**DOI:** 10.3390/foods14091653

**Published:** 2025-05-07

**Authors:** Longquan Xiao, Lin Cui, Molazi Lapu, Ting Bai, Juan Wang, Xiaoying Guo, Dayu Liu, Mingxue Liu, Xinhui Wang

**Affiliations:** 1 School of Food and Biological Engineering, Chengdu University, Chengdu 610106, China; xiaolongquan@cdu.edu.cn (L.X.);; 2 School of Life Science and Engineering, Southwest University of Science and Technology, Mianyang 621010, China; 3 Yibin Etiange Food Co., Ltd., Yibin 644100, China; 4 Chongqing Academy of Metrology and Quality Inspection, Chongqing 400020, China; 5 Sichuan Kelun Pharmaceutical Co., Ltd., Chengdu 610599, China

**Keywords:** goat meat, microbial community, assembly processes, chilled storage

## Abstract

Microbial community succession is closely related to the corruption of meat, but there are few studies on microbial community assembly and their relationship with physicochemical indexes in meat during chilled storage (4 °C). This study aimed to investigate the mechanism of bacterial community assembly and the effect of microbial succession on quality changes during the preservation of goat meat. The results showed that the stochastic process was the primary driving mechanism during community construction. During the chilled storage, the predominant bacteria in the three groups at the genus level were *Acinetobacter* and *Pseudomonas*. With the extension of storage duration, the relative abundance of *Pseudomonas* in samples from local markets and slaughterhouses increased rapidly and gradually acted as dominant flora during the succession process. Spearman correlation analysis revealed that *Pseudomonas* exhibited a highly significant positive association with total volatile basic nitrogen (TVB-N) and a highly significant negative correlation with redness (*p <* 0.01), which is crucial in the degradation of meat quality. These results provide guidance for regulating the microbial communities of goat meat during preservation by optimizing the storage conditions to delay the deterioration of goat meat.

## 1. Introduction

In comparison to other conventional red meats, goat meat has more unsaturated fatty acids and less total fat, which appeals to consumers who are health-conscious [1]. However, livestock and poultry meat are inevitably contaminated by microorganisms during the slaughtering, transportation, and marketing stages, which eventually leads to problems such as browning in color and a short shelf life [2,3]. According to studies, the primary factors influencing the deterioration of chilled meat are the development and metabolism of bacteria [4]. Under refrigeration conditions, some microorganisms can adapt to special environments and become dominant microorganisms [5,6]. The most common bacteria found in chilled meat under aerobic refrigeration include *Pseudomonas*, *Acinetobacter*, *Brochothrix*, *Shewanella*, *Aeromonas*, etc. [7,8,9], with the change in the preservation conditions and the type of meat, the dominant microorganisms will change accordingly [10,11].

In the last decade, reports of bacterial community succession in refrigerated pork, beef and lamb have been widespread [9,12,13,14,15], but reports of the mechanism of microbial community assembly in meat during chilled storage are very rare. So, the intricate mechanisms underlying the establishment and evolution of microbial community structures in meat during chilled storage require further elucidation. Nowadays, two ecological processes, the deterministic processes and stochastic processes are considered to be the rules regulating the assembly of bacterial communities [16,17]. The deterministic processes highlight the preeminent influence of environmental selections on microbial community assembly, while stochastic processes suggest that microbial taxa coexist in overlapping environmental niches rather than eliminate each other, suggesting that competitive ability matched and random variations are unrelated to environmental adaptability [18].

In this research, the assembly processes and co-occurrence network of bacterial communities were analyzed during preservation of goat meat, and the Spearman rank correlation analysis was used to analyze the relationship between the bacterial communities and TVB-N, color, total plate count, mass loss rate, pH, and myoglobin, and comprehensively evaluated the effect of bacterial communities on the quality changes in goat meat during preservation. This work offers a theoretical basis for the quality prediction of goat meat and the improvement of chilled goat meat storage and transportation technology.

## 2. Materials and Methods

### 2.1. Sample Collection and Pretreatment

The goat meat samples (Longissimus thoracis et lumborum) were from three different goats, the samples of three Boer goats were transferred to the laboratory within two hours of purchase and named BA (Boer goat was purchased from agricultural trade market, distance and time from the market to the laboratory were 2 km and 20 min, respectively), BS (Boer goat was purchased from slaughterhouse, and the distance and time from the slaughterhouse to the laboratory were 32 km and 70 min, respectively) and BM (Boer goat was purchased from the local market, and the distance and time from the market to the laboratory were 4 km and 30 min, respectively). Goat meat samples were transported using ice packs for temperature control. The goat meat was cut into pieces of about 10 g in size, and every three pieces of goat meat were placed in a sterile disposable Petri dish, before all samples were placed in a refrigerator (4 °C), and all experiments were performed in three technical replicates.

### 2.2. Determination of pH

A portable pH meter (Testo205 portable pH meter, Shenzhen Testo Instrument Co., Ltd., Shenzhen, China) was used to directly insert the samples to detect the pH, and the points of three samples were randomly picked for each measurement [19]. The pH of the samples was measured during different storage times (1, 2, 3, 4, 5, 6, and 7 days).

### 2.3. Determination of Color Value

The Red–Green Axis (a*) of the samples was measured by a colorimeter (CS-220, Hangzhou Color Spectrum Technology Co., Ltd., Hangzhou, China), and the points of the three samples were randomly picked for each measurement. The a* of the samples was carried out at different storage times (1, 2, 3, 4, 5, 6, and 7 days).

### 2.4. Determination of Mass Loss Rate

The mass loss rate (ML) of the samples was measured in triplicate at different storage times (2, 3, 4, 5, 6, and 7 days). The mass loss rate of the samples was determined using the following equation.

Mass loss (%) = (m_0_ − m_1_)/m_0_ (1)

where m_0_ is the weight of the samples at the beginning of preservation, g; and m_1_ is the weight of the samples after preservation at 4 °C, g.

### 2.5. Determination of Myoglobin

The myoglobin was determined by the method provided by Krzywicki and Karol with a slight modification [19], and the myoglobin analyses were performed in triplicate at different storage times (1, 2, 3, 4, 5, 6, and 7 days). Briefly, goat meat samples (5 g) were homogenized in 25 mL of phosphate buffer (0.04 M, pH 6.8), the homogenized samples were incubated at 4 °C for 1 h in darkness and subsequently centrifuged (4500× *g*, 20 min, 4 °C). The supernatant was filtered through filter paper, and the absorbances of filtrate at 525, 545, 565, and 572 nm were measured with a spectrophotometer (UV-5200 UV-Vis Spectrophotometer, Shanghai Yuanxi Instrument Co., Ltd., Shanghai, China), respectively. The calculation formulas of deoxymyoglobin (DMb), oxymyoglobin (OMb), and metmyoglobin (MMb) are as follows:

DMb = (0.369 × R1 + 1.140 × R2 − 0.941 × R3 + 0.015) × 100(2)

OMb = (0.882 × R1 − 1.267 × R2 + 0.809 × R3 − 0.361) × 100(3)

MMb = (−2.514 × R1 + 0.777 × R2 + 0.800 × R3 + 1.098) × 100(4)

where R_1_, R_2_, R_3_ are absorbance ratios A572 nm/A525 nm, A565 nm/A525 nm, A545 nm/A525 nm.

### 2.6. Determination of Total Volatile Basic Nitrogen (TVB-N)

The TVB-N was determined according to the Chinese Standard [20], and the TVB-N analyses were performed in triplicate at different storage times (1, 2, 3, 4, 5, 6, and 7 days). In short, 100 mL of trichloroacetic acid solution at a concentration of 20 g/L and 10 g of minced goat meat were combined in a conical flask, incubated for 30 min, and then filtered. Subsequently, 5 mL of MgO (10 g/L) was added to the reaction chamber containing 10 mL of the filtrate, and the glass cap was promptly secured for distillation. The receiving liquid was titrated with HCl (0.0100 mol/L) until the endpoint was reached.

### 2.7. Determination of Total Plate Count (TPC)

The TPC of the sample was determined by the method in the Chinese Standard [21], and the TPC analyses were performed in triplicate at different storage times (1, 2, 3, 4, 5, 6, and 7 days). Approximately 10 g of each goat meat sample was placed in a sterile homogenization bag with 90 mL of sterile saline solution (NaCl, 0.85%), then the sample was homogenized for 2 min by a Stomacher paddle homogenizer to obtain the stock solution. The stock solution was serially diluted and counted using the surface plate method. The TPC of goat meat log_10_ (CFU/g) was determined.

### 2.8. DNA Extraction and Bacterial Community Analysis

Bacterial communities were detected by high-throughput sequencing using primers (341F: 5′-CCTACGGGRBGCASCAG-3′, 806R: 5′-GGACTACNNGGGTATCTAAT-3′) to amplify the V3-V4 region of the 16S rRNA gene. All PCR reactions were carried out with 15 μL of Phusion^®^ High-Fidelity PCR Master Mix (New England Biolabs, Ipswich, MA, USA); 0.2 μM of each primer and 10 ng of target DNA. PCR amplification was conducted using the following procedure: first denaturation step at 98 °C (1 min), followed by 30 cycles at 98 °C (10 s), 50 °C (30 s), and 72 °C (30 s); a concluding extension at 72 °C for 5 min. The PCR results were analyzed using 2% agarose gel electrophoresis and subsequently purified with the Qiagen Gel Extraction Kit (Qiagen, Hilden, Germany). In accordance with the manufacturer’s guidelines, the sequencing libraries were constructed with the NEBNext^®^ UltraTM IIDNA Library Prep Kit (Ipswich, England), and their quality was assessed using the Qubit^®^ 2.0 Fluorometer (Thermo Scientific, Waltham, MA, USA) and the Agilent Bioanalyzer 2100 system (Santa Clara, CA, USA). The library was ultimately sequenced using an Illumina NovaSeq (San Diego, CA, USA) technology, producing 250 bp paired-end reads. High-quality sequences were acquired following paired-end read assembly and quality checking [21,22,23], then assembled and applied to operational taxonomic unit (OTU) and taxonomic analysis [24]. The alpha diversity indices were calculated by QIIME2 software to determine the complexity of species among groups [25]. The samples from each goat were collected at different storage times (1, 4, and 7 days) in triplicate.

### 2.9. Models of Microbial Community Assembly

To evaluate community assembly processes, the *β*-nearest taxon index (*β*NTI) and Raup-Crick Bray–Curtis index (RC_bray_) of the bacterial community during the chilled storage were calculated by the method described by Stegen et al. [26,27]. Generally, if *β*NTI > 2, it suggests that variable selection or heterogeneous selection predominated community succession, while *β*NTI < −2 indicates the dominance of homogenous selection. |*β*NTI| < 2 means the community assembly was regulated by a stochastic process rather than a deterministic process. If |*β*NTI| < 2 and RC_bray_ > 0.95, the difference between communities was attributed to dispersal limitation, and RC_bray_ < −0.95 indicates that community succession was influenced by homogenizing dispersal. If |RC_bray_| < 0.95, community heterogeneity was due to undominated processes.

### 2.10. Statistics Analysis

The data were expressed as means ± standard deviation. Analysis of variance (ANOVA) was performed using SPSS statistical software (Version 27.0, SPSS Inc., Chicago, IL, USA), with *p* < 0.05 considered significant differences, and Duncan’s test was used for comparison of means.

The correlation matrices were plotted by the corrplot package in R language (Version 4.0.2), and the correlation coefficients between bacterial communities and physicochemical properties were computed using the Spearman method, respectively.

## 3. Results and Discussion

### 3.1. Quality Changes in Goat Meat During Chilled Storage

**Figure 1:**
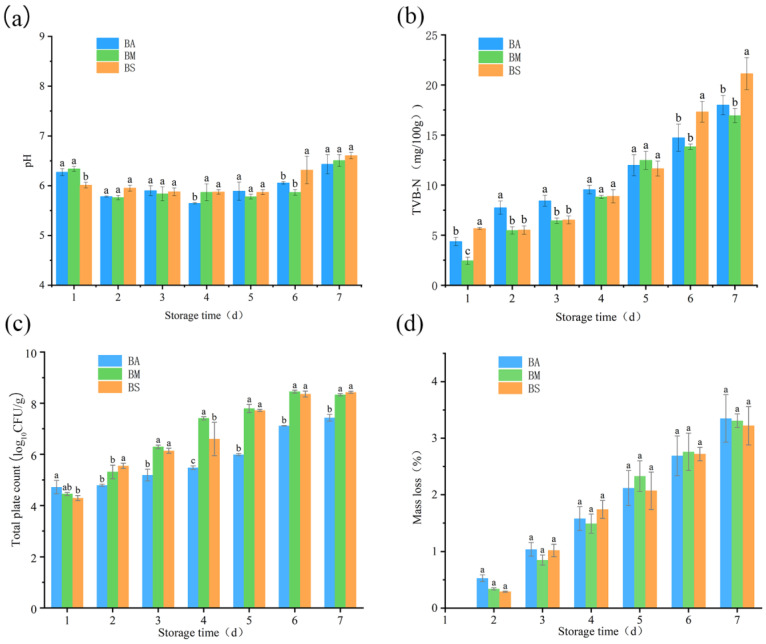
Changes in pH (**a**), TVB-N (**b**), TPC to log_10_ count (**c**), and mass loss (**d**) of goat meat during preservation at 4 °C. Different letters in each column indicate significant differences at *p* < 0.05. BA: the fresh samples of Boer goat purchased from the agricultural trade market, BM: the fresh samples of Boer goat purchased from the local market, the same below, BS: the fresh samples of Boer goat purchased from a slaughterhouse, the same below.

The pH, TVB-N, TPC, and mass loss rate of goat meat were measured to evaluate the quality changes in meat during preservation (4 °C). The pH has a significant impact on changes in meat quality, and it can affect meat color by influencing metmyoglobin reductase activity and oxygen consumption [22]. It can be seen from Figure 1a that the initial pH of the three groups ranged from 6.01 to 6.34. From the 1st to 7th day of preservation, the pH of group BA showed an upward trend and increased rapidly after the 4th day. Combined with Figure 1b, it can be seen that this may be related to the accumulation of TVB-N. On the 2nd day of preservation, the pH of the three goat meat samples decreased, which was mainly caused by the production of lactic acid through the glycolysis [23].

TVB-N is a crucial indicator for assessing the freshness of chilled meat, and it is produced by the degradation of proteins [24,25]. As shown in Figure 1b, the initial TVB-N values of BA, BM, and BS were 4.37 mg/100 g, 2.44 mg/100 g, and 5.67 mg/100 g, respectively. On the 7th day of the storage, the TVB-N of group BS was 21.13 mg/100 g, and that of group BA and BM were 17.99 mg/100 g and 16.93 mg/100 g, respectively. In accordance with the Chinese Standard [26], fresh goat meat should have a TVB-N value less than 15 mg/100 g; if the TVB-N of the meat exceeds this value, it means the meat has been spoiled. Therefore, it can be concluded that the meat quality of the samples from BS has been spoiled on the 6th day of preservation, and the groups of BA and BM have reached the spoilage standard on the 7th day of preservation.

As shown in Figure 1c, from the 1st to 7th days of the storage, the total plate counts (TPC) increased with the increase in preservation time. In accordance with the Chinese Standard [27], the TPC of fresh goat meat must be below 6 log_10_ CFU/g; on the 4th day of preservation, the samples from BM and BS reached the spoilage standard, and the samples from group BA reached the spoilage standard on the 5th day of preservation. As shown in Figure 1d, the mass loss rate (ML) of BA, BM, and BS on the second day of preservation was 0.53%, 0.34%, and 0.29%, respectively. During the whole preservation period, the ML of the three sample groups exhibited a consistent increasing trajectory, with no significant differences seen among the ML values of the groups.

### 3.2. Red–Green Axis (a*) and Myoglobin Changes in Goat Meat During Chilled Storage

**Figure 2:**
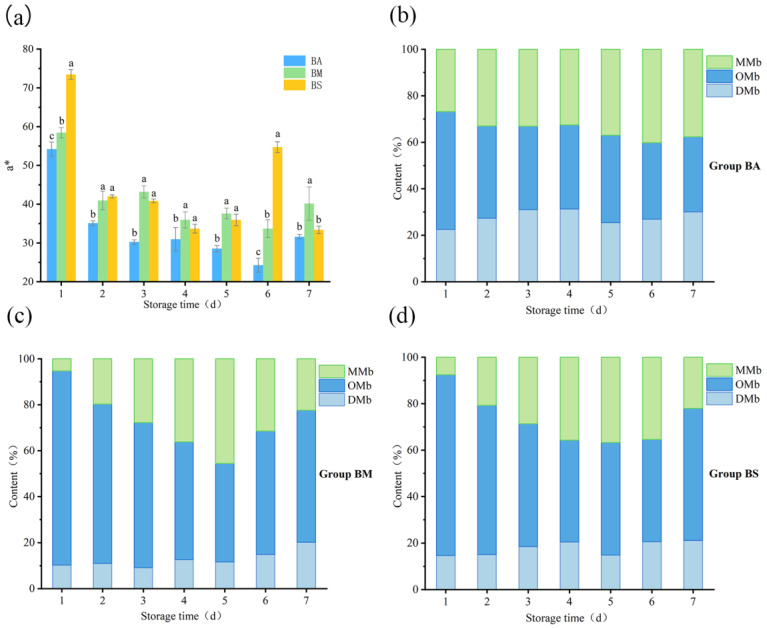
Changes in a* (**a**) and myoglobin (**b**–**d**) in different groups of goat meat during storage at 4 °C. DMb, OMb, and MMb are deoxy-myoglobin, oxy-myoglobin, and met-myoglobin, respectively.

Color is a critical indicator for meat, and it significantly affects the customer’s purchase decision. The a* value represents the Red–Green axis, which ranges from −128 to +128. A positive value of a* skews the color towards red, while a negative value of a* skews the color towards green. When a value is positive, the larger the value, the redder the color is; when a value is negative, the smaller the value, the greener the color is. The initial a* of BA, BM, and BS was 54.20, 58.45, and 73.46 on the 1st day of preservation (Figure 2a), respectively. On the 2nd day of preservation at 4 °C, the a* of the three groups decreased significantly, indicating that the color of the goat meat changed rapidly after slaughter. However, the a* of the three groups increased to varying degrees after the 4~6 days of preservation, which was consistent with the changing trend of oxymyoglobin (OMb) in the three groups (Figure 2b–d). As shown in Figure 2, the OMb was mainly derived from the reduction in metmyoglobin (MMb), which was due to the increased activity of myoglobin reductase with the increase in pH [28]. Ultimately, the increase in OMb led to an increase in the a* value of the samples.

### 3.3. Microbial Richness and Diversity of Goat Meat During Chilled Storage

The α-diversity analysis can show the abundance and diversity of microflora. The coverage index of all samples was greater than 0.999, which indicated that the sequencing results can reflect the real situation [29]. Pielou_e index can reflect the evenness of species; the more uniform the species, the greater the Pielou_e [30]. The Chao1 index is typically employed to indicate the species abundance of a community; the more species with low relative abundance in the community, the greater the Chao1 index. The Shannon and Simpson indices reveal the species diversity of the community; the higher the species diversity, the greater the Shannon and Simpson indices [15,31].

As demonstrated in Table 1, the Shannon and Pielou_e index of three groups decreased significantly on the 4th day during the chilled storage (*p* < 0.05), indicating that species diversity and evenness decreased during this period, which may be due to the low temperature, resulting in some microorganisms stopping their activities, leading a significant decrease in Shannon and Pielou_e index. On the 7th day of storage, the Chao1, Shannon, Simpson, and Pielou_e indices of the BS group continued to decline, while those of the BM group rebounded, which may be due to differences in microbial communities, leading some microbial to adapt to the low-temperature environment, resulting in an increase in species richness and uniformity.

### 3.4. Analysis of Microbial Community Dynamics During Chilled Storage

**Figure 3:**
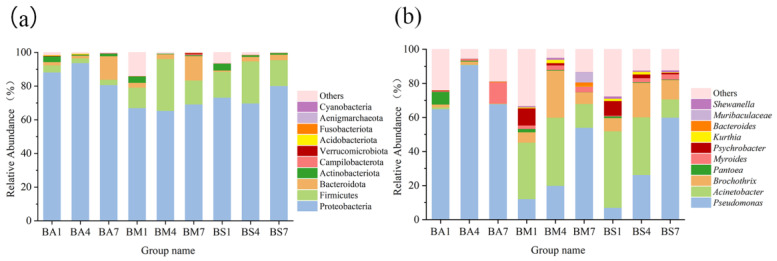
Changes in bacterial communities at phyla level (**a**) and the genus level (**b**). BA1, BA4, BA7 represent the samples collected from group BA on the 1st, 4th, and 7th day of the preservation, groups BM and BS were named according to the same rules, the same as below.

Proteobacteria and Firmicutes were dominant phyla in three groups during the chilled storage, and similar results were reported elsewhere (Figure 3a) [13]. These two types of bacteria are the main flora in the gut of goat [32], and it can be guessed that the microbial contamination in goat meat may mainly come from the slaughtering process, which aligns with the conclusion of Wolfe that environmental microorganisms play a critical role in the formation of food microbiome [33]. From the 1st to the 4th day of preservation, the relative abundance of Proteobacteria in BM and BS groups decreased slightly, whereas Firmicutes’ relative abundance rose during this time. On the 7th day of the storage, Proteobacteria’s relative abundance in BM and BS groups rebounded, while Firmicutes’ relative abundance decreased significantly, and the relative abundance of Bacteroidota increased significantly.

At the level of genus, *Pseudomonas*, *Acinetobacter,* and *Brochothrix* were the dominant bacteria in the chilled storage (Figure 3b), which is similar to many reported studies [34,35,36]. From the 1st day to the 7th day of the storage, the relative abundance of *Pseudomonas* in BM and BS groups increased rapidly and gradually became the dominant bacteria. The *Brochothrix*’ relative abundance increased notably on the 4th day and decreased significantly on the 7th day of chilled storage, indicating that the succession of microbial community in goat meat was mainly influenced by the storage environment. Studies have shown that the extracellular enzymes secreted by *Pseudomonas* have strong protease activity on myostromin, which helps the bacteria penetrate the meat, obtain new sources of nutrients, soften the meat and increase the formation of meat mucus [37,38], and *Brochothrix* is closely related to meat softening and mucus formation [39]. To preserve the freshness of goat meat, it is very important to prevent the growth of *Pseudomonas* and *Brochothrix*.

### 3.5. Ecological Assembly Processes of Bacterial Communities

**Figure 4:**
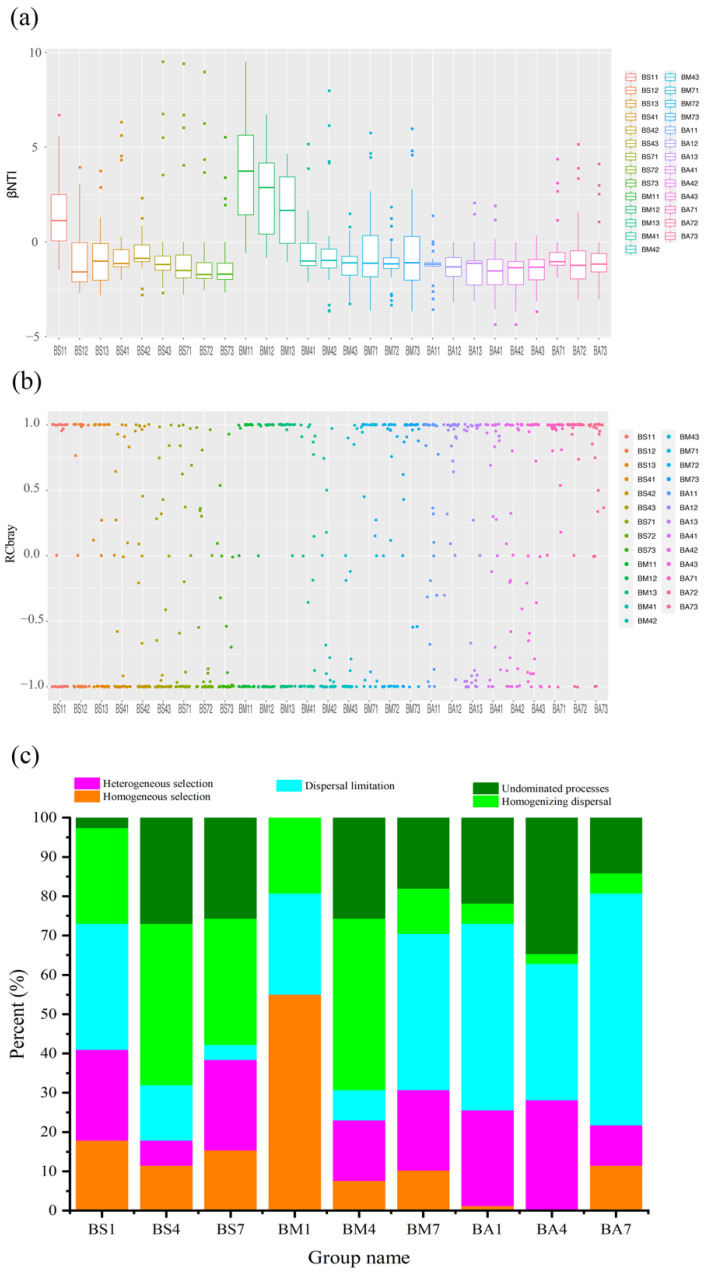
Ecological processes that generate the community succession patterns during the preservation process of goat meat. (**a**) *β*NTI results of the bacterial community; *β*NTI > 2, heterogeneous selection; *β*NTI < −2, homogeneous selection; |βNTI| < 2, stochastic process. (**b**) RC_bray_ results of the bacterial community; RC_bray_ > 0.95, dispersal limitation; RC_bray_ < −0.95, homogenizing dispersal; |RC_bray_| < 0.95, undominated processes, which is the same as below. (**c**) The relative contribution rate of ecological succession during community construction during goat meat preservation.

To analyze the assembly processes of microbial communities during the chilled storage of goat meat, firstly the change in βNTI index was calculated based on microbial community assembly theories [40,41]. As shown in Figure 4a, the stochastic process (|*β*NTI| < 2) dominated the bacterial community assembly, indicating the bacteria in goat meat initially comes from the process of slaughtering, packaging, and transportation, which formed stable microorganism community during long-term operation as described in other studies [42]. Compared with deterministic processes, stochastic processes can produce more diverse ecological functions and buffer the disturbance caused by environmental changes, which is conducive to maintaining the stability and sustainability of ecosystem functions [43,44,45].

With the preservation in a relatively stable chilled storage environment, microorganisms erupt and inhibit their growth due to different adaptability, and they gradually form a microbial community structure dominated by spoilage bacteria, so the stochastic process (|*β*NTI| < 2) was the main driving mechanism for this community construction during chilled storage of goat meat. The results of RC_bray_ index (Figure 4b,c) showed that in the stochastic process, homogenizing dispersal (RC_bray_ < −0.95) and dispersal limitation (RC_bray_ > 0.95) were the main microbial community formation process, which may be due to that the goat meat was preserved in a relatively constant temperature environment (4 °C) with less influence from other environmental factors [46]. Therefore, the stochastic process in the assembly of the bacterial communities during the chilled storage of goat meat suggests that microbial control in the slaughtering and storage stages is critical.

### 3.6. Co-Occurrence Networks Revealed Bacterial Interactions at Genus Level

**Figure 5:**
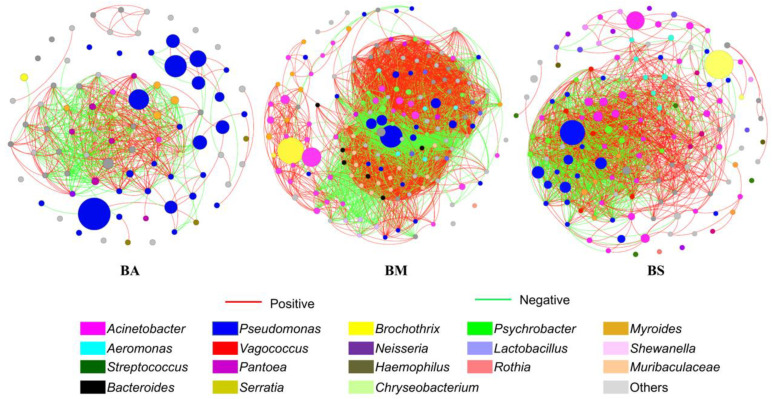
Co-occurrence networks revealed bacterial interactions at genus level. The size of each node is proportional to the relative abundance of bacterial genera.

In this research, the modularity index was all larger than 0.48 (Table 2), which indicated that the co-occurrence network of these three co-occurrence networks had modular profiles. *Pseudomonas* dominated the network interactions of the three groups, with the highest number of inter-connections with other species (Figure 5). The proportions of positively correlated connections in the co-occurrence network of BA, BM, and BS groups were 58.86%, 59.49% and 63.01%, respectively (Table 2), which indicated that there was a tendency towards cooperative rather than competitive relationships among bacteria. This more complex network is considered to be the result of environmental selection, which is consistent with the previous calculation results [46,47]. So, in the process of chilled storage of goat meat, *Pseudomonas* microorganisms have strong competitiveness in the preservation process and gradually become the dominant spoilage strain. This has important reference significance for the bacteriostatic control of goat meat during preservation in the future.

### 3.7. Analysis of the Relationship Between Bacterial Community and Physicochemical Indexes During Chilled Storage

**Figure 6:**
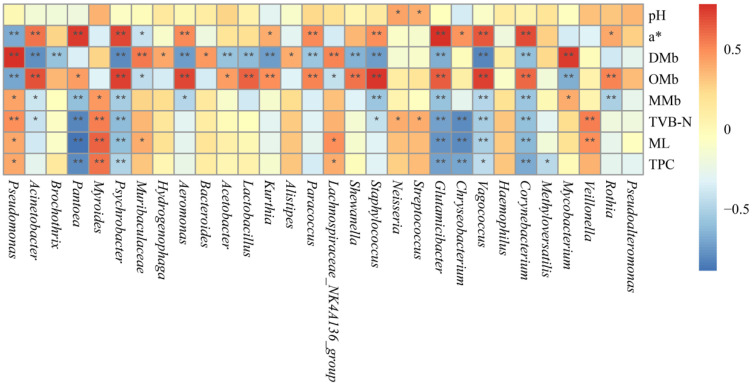
Spearman correlation analysis between bacteria and physicochemical indexes of chilled goat meat. The significant correlation was marked with asterisk: * 0.01 < *p* ≤ 0.05, ** 0.001 < *p* ≤ 0.01.

The relationship between the physiochemical indexes and the bacteria of the top 30 genera at the genus level were investigated with Spearman correlation analysis. As shown in Figure 6, *Pseudomonas* is significantly positively correlated with TVB-N, DMb, ML, and TPC, and it is extremely significantly negatively correlated with a* (*p* < 0.01); it can be seen that during the storage process, *Pseudomonas* was the dominant flora and performed a critical role in the spoilage of meat. In addition, *Myroides*, *Neisseria*, *Streptococcus*, *Veillonella* and TVB-N were also positively correlated. Above all, it can be seen that *Pseudomonas* contribute significantly to the browning of meat color, which is similar to the browning of the other red meat [35].

## 4. Conclusions

The main conclusions are as follows: (1) Stochastic processes (dispersal limitation and homogenizing dispersal) were the main driving mechanisms for community construction. (2) With the increase in chilled storage time, *Pseudomonas* gradually evolved into the predominant flora, *Pseudomonas*, *Myroides*, *Neisseria*, etc., which play a critical role in the deterioration of fresh meat. (3) *Pseudomonas* exhibits a significantly positive correlation with TVB-N and an exceedingly strong negative correlation with a* (*p* < 0.01). This study can provide a theoretical basis for predicting the shelf life of chilled goat meat and improving the preservation technology of goat meat.

## Figures and Tables

**Table 1:** General information of sequence and alpha diversity analysis of goat meat during chilled storage.

Samples	Chao1	Coverage	OTUs	Pielou_e	Shannon	Simpson
BA1	501.31 ± 211.37 ^a^	1.0000 ^a^	499 ± 211 ^a^	0.5317 ± 0.00 ^b^	4.72 ± 0.35 ^ab^	0.883 ± 0.003 ^b^
BA4	348.96 ± 48.54 ^a^	1.0000 ^a^	345 ± 49 ^a^	0.4767 ± 0.01 ^c^	4.01 ± 0.20 ^c^	0.886 ± 0.010 ^b^
BA7	342.99 ± 87.97 ^a^	1.0000 ^a^	342 ± 88 ^a^	0.5980 ± 0.04 ^a^	5.02 ± 0.52 ^a^	0.932 ± 0.016 ^a^
BM1	543.72 ± 33.95 ^a^	0.9993 ^a^	537 ± 38 ^a^	0.7267 ± 0.01 ^a^	6.59 ± 0.15 ^a^	0.975 ± 0.003 ^a^
BM4	215.64 ± 25.70 ^c^	1.0000 ^a^	207 ± 24 ^c^	0.5797 ± 0.02 ^b^	4.46 ± 0.28 ^c^	0.881 ± 0.021 ^b^
BM7	371.08 ± 9.32 ^b^	1.0000 ^a^	365 ± 13 ^b^	0.5850 ± 0.03 ^b^	4.98 ± 0.23 ^b^	0.899 ± 0.025 ^b^
BS1	443.70 ± 9.71 ^a^	0.9993 ^a^	439 ± 9 ^a^	0.7200 ± 0.01 ^a^	6.32 ± 0.08 ^a^	0.972 ± 0.003 ^a^
BS4	274.42 ± 40.33 ^b^	0.9997 ^a^	266 ± 38 ^b^	0.6317 ± 0.02 ^b^	5.08 ± 0.26 ^b^	0.920 ± 0.022 ^b^
BS7	231.51 ± 32.68 ^b^	1.0000 ^a^	227 ± 31 ^b^	0.6010 ± 0.04 ^b^	4.70 ± 0.42 ^b^	0.908 ± 0.024 ^b^

Notes: All data are shown as the mean (*n* = 3) ± standard deviation. Distinct letters in the same group mean significant difference at *p* < 0.05. BA1, BA4, BA7 represent the samples collected from group BA on the 1st, 4th, 7th day of the preservation, groups BM and BS were named according to the same rules, and the same below.

**Table 2:** Main topological features of microbial community’s co-occurrence networks during chilled storage of goat meat.

Name	Group Name		
	BA	BM	BS
Number of nodes	85	165	144
Number of edges	193	1283	218
Positive (%)	58.86	59.49	63.01
Negative (%)	41.14	40.51	36.99
Average degree	4.541	15.552	3.028
Average path length	1.260	1.570	2.784
Network diameter	2.841	5.445	6.616
Clustering coefficient	0.866	0.743	0.567
Graph density	0.054	0.095	0.021
Centralization betweenness	0.0059	0.0066	0.0252
Centralization degree	0.148	0.143	0.091
Connectance	0.054	0.095	0.021
Modularity	0.486	0.505	0.625

## Data Availability

The data presented in this study are available upon request from the corresponding author.
